# Mean Arterial Pressure Is Related to Incident Nonalcoholic Fatty Liver Disease among the Nonobese Female with Normal Low-Density Lipoprotein Cholesterol Levels: A Large Cohort Study in China

**DOI:** 10.1155/2020/3580840

**Published:** 2020-01-17

**Authors:** Shangbo Xu, Lan Chen, Danhua Hong, Lihua Yang, Xiaozhi Li, Xin Wang

**Affiliations:** ^1^Department of Cardiology, First Affiliated Hospital of Shantou University Medical College, No. 57 of Changping Road, Shantou, Guangdong 515041, China; ^2^Department of Nephrology, First Affiliated Hospital of Shantou University Medical College, No. 57 of Changping Road, Shantou, Guangdong 515041, China; ^3^Department of Oncology, First Affiliated Hospital of Shantou University Medical College, No. 57 of Changping Road, Shantou, Guangdong 515041, China

## Abstract

**Aim:**

We aimed to demonstrate the independent effect of mean arterial pressure (MAP) on incident nonalcoholic fatty liver disease (NAFLD) among the nonobese Chinese with normal low-density lipoprotein cholesterol (LDL-C) levels.

**Methods:**

16,153 nonobese participants without NAFLD at baseline were enrolled and then assigned to four groups by quartiles of MAP (Q1-Q4). A subgroup analysis by gender was also conducted. Participants were diagnosed with NAFLD by ultrasonography.

**Results:**

During a mean follow-up of 2.80 years, the cumulative incidence of NAFLD was 14.37 and the incidence rate was 513.17 per 10,000 person-years. The cumulative incidence of NAFLD for the whole population or gender groups gradually increased with the quartiles of MAP (all *P* < 0.001). In the Q4 of MAP, the cumulative incidence of NAFLD for the whole population, male, and female reached up to 6.22 (5.75-6.70), 6.70 (6.21-7.19), and 5.69 (5.24-6.14), respectively. After adjustment for potential confounders, as compared with Q1, the hazard ratio for NAFLD was 1.328 (1.072-1.647), 1.625 (1.276-2.069), and 1.697 (1.231-2.340) for Q2, Q3, and Q4, respectively. In subgroup analysis, the respective hazard ratio for NAFLD in Q2, Q3, and Q4 of MAP was 1.760 (1.276-2.429), 2.080 (1.433-3.019), and 2.377 (1.452-3.890), compared with female in the Q1 of MAP. But MAP was not associated with incident NAFLD in male. Besides, MAP had a larger area under the receiver-operating characteristic curves than SBP or DBP, with optimal cutoff point of 88 mmHg in male and 89 mmHg in female.

**Conclusions:**

MAP is an independent predictor for incident NAFLD among nonobese female with normal LDL levels.

## 1. Introduction

Nonalcoholic fatty liver disease (NAFLD) is recognized as a common spectrum liver disease, encompassing nonalcoholic fatty liver (simple steatosis) and nonalcoholic steatohepatitis (NASH), which may progress to cirrhosis even hepatocellular carcinoma at last [1]. Owing to the epidemic of obesity, the prevalence of NAFLD has been increasing dramatically and was reported approximately 25–30% of the general population worldwide [1, 2], including Asia–Pacific region which was thought to be a nonepidemic area in the past [3]. Furthermore, it has been demonstrated that NAFLD is a significant risk factor for developing hypertension, type 2 diabetes mellitus, and cardiovascular disease [4–6]. Therefore, it is quite interesting to identify individuals at a high risk of developing NAFLD.

Blood pressure (BP), including systolic blood pressure (SBP) and diastolic blood pressure (DBP), is an important risk factor for developing NAFLD [7–10], even in individuals without hypertension [10]. Mean arterial pressure (MAP), a steady component of BP, has wildly been used in clinical practice. However, no study is available with respect to the association between MAP and incident NAFLD. For another, most previous studies paid their attention to progression of NAFLD among the obese population. And a previous study enrolled 183,903 nonobese Chinese with normal low-density lipoprotein cholesterol (LDL-C) levels, of which 25,503 individuals were diagnosed with NAFLD by ultrasonography [11].

Our study therefore is aimed at demonstrating the association between MAP and incident NAFLD among a nonobese population with normal LDL-C levels in China.

## 2. Materials and Methods

### 2.1. Study Design and Participants

The raw data that we tried to analyze was downloaded freely from an online database named “DATADRYAD” (http://www.Datadryad.org), which supports the reuse of data of published studies. In the light of terms of service, we cited the data package upload by Sun et al. in our present study [11, 12]. We performed a secondary analysis with no need for another ethic approval because the original study protocol was vetted and approved by the ethics committee of Wenzhou People's Hospital. The participants in the original study were individuals who attended a health examination from January 2010 to December 2014. Participants would undergo annual evaluation throughout the follow-up period. Individuals were excluded if they met the following criteria: (1) excess consumption of alcohol (more than 140 g/week for male and 70 g/week for female); (2) any known causes of chronic hepatic disease, such as viral hepatitis or autoimmune hepatitis; (3) a body mass index (BMI) equal to or more than 25 kg/m^2^; (4) a high level of LDL-C (>3.12 mmol/L); (5) those taking antihypertensive, antidiabetic, or lipid-lowering agents; and (6) lost to follow-up or with missing data. At the same time, we eliminated 20 participants without available SBP and/or DBP. Finally, a total of 16,153 participants without NAFLD at the baseline were included in our research. The process of the selection was specifically exposited in the previous report [11].

### 2.2. Data Collection

The following variables included in the data package were extracted: gender, age, body mass index (BMI), SBP, DBP, alkaline phosphatase (ALP), alanine aminotransferase (ALT), aspartate aminotransferase (AST), albumin (ALB), globulin (GLB), total bilirubin (TB), blood urea nitrogen (BUN), creatinine (Cr), uric acid (UA), fasting plasma glucose (FPG), total cholesterol (TC), triglyceride (TG), high-density lipoprotein cholesterol (HDL-C), LDL-C, duration of follow-up, and outcome of follow-up. The trained medical staff used a standardized procedure to obtain participant's medical history and health habit. BP was measured with an automated sphygmomanometer with the subjects in a sitting position and a quiet environment. Fasting venous blood sample collection (required an overnight fast) would be measured with an automated analyzer (Abbott AxSYM). More specific details were presented in the previous reports [8, 11].

### 2.3. Definitions and Outcomes

BMI was calculated as the weight (kg) to the height (m^2^), and MAP was calculated as [SBP + (2 × DBP)]/3. Participants were diagnosed with NAFLD by ultrasonography according to the recommendations of the Chinese Liver Disease Association [13]. Generally speaking, NAFLD was diagnosed as diffuse enhancement of the close field echo in the hepatic region (greater than in the region of the kidney and spleen) and gradually attenuated beam in the far field echo, if in combination with one of the following items: intrahepatic lacuna structure is unclearly displayed; a round and blunt border in mild-to-moderate hepatomegaly; a decrease of the blood flow signal, while the distribution of blood flow is still normal; and the display of envelop of the right liver lobe and diaphragm is unclear or nonintact.

### 2.4. Statistical Analysis

All participants were classified into four groups based on the quartiles of baseline MAP: Q1 (58 to <80 mmHg), Q2 (80 to <88 mmHg), Q3 (88 to <96 mmHg), and Q4 (96 to ≤142 mmHg). Continuous variables were expressed as mean ± standard deviation (SD), while categorical variables were expressed as number (percentage). The one-way ANOVA and chi-squared test were used to determine any statistical differences of baseline characteristics for the continuous and categorical variables, respectively. Meanwhile, differences of baseline data between male and female were performed. Then, we calculated the incidence rate (per 10,000 person-years) and cumulative incidence with 95% confidence interval (CI) of NAFLD. Cumulative hazard curves were plotted using the Kaplan-Meier method, and the log-rank test was used to compare the cumulative incidence of NAFLD stratified by MAP. We performed the cox's proportional hazard regression analyses to evaluate the association of MAP with the incident NAFLD. Hazard ratio (HR) with a 95% CI was reported. We also conducted a subgroup analysis stratified by gender. Receiver-operating characteristic (ROC) curve was used to compare the abilities of SBP, DBP, and MAP in predicting the incident NAFLD. The optimal cutoff of MAP was calculated according to the highest Youden index (sensitivity+specificity-1). A *P* < 0.05 (two tailed) was considered statistically significant. All analyses involved the use of SPSS V.25.0 software (SPSS Inc., Chicago, Illinois, USA).

## 3. Results

### 3.1. Baseline Characteristics of Participants

We included a total of 16,153 participants (8,472 men, 52.4%) who were free of NAFLD at baseline. The mean age of this population was 43.23 years, and the MAP ranged from 58 to 142 mmHg. The baseline characteristics of participants by MAP quartiles are shown in Table 1. Age, the number of males, BMI, SBP, DBP, ALP, ALT, AST, ALB, GLB, TB, BUN, Cr, UA, FPG, TC, TG, and LDL-C all tended to be higher in the higher MAP compared with the lower MAP (*P* < 0.001), but HDL-C changed in the opposite trend simultaneously (*P* < 0.001). Then, an analysis stratified by gender was also conducted and is summarized in Table 2. Compared with female, male was older, with higher BMI, SBP, DBP, ALP, ALT, AST, ALB, TB, BUN, Cr, UA, FPG, TG and the incidence rate of NAFLD, and lower HDL-C. However, no significant differences were observed in MAP, GLB, TC, and LDL-C between female and male.

### 3.2. Incidence Rate of NAFLD

**Figure 1:**
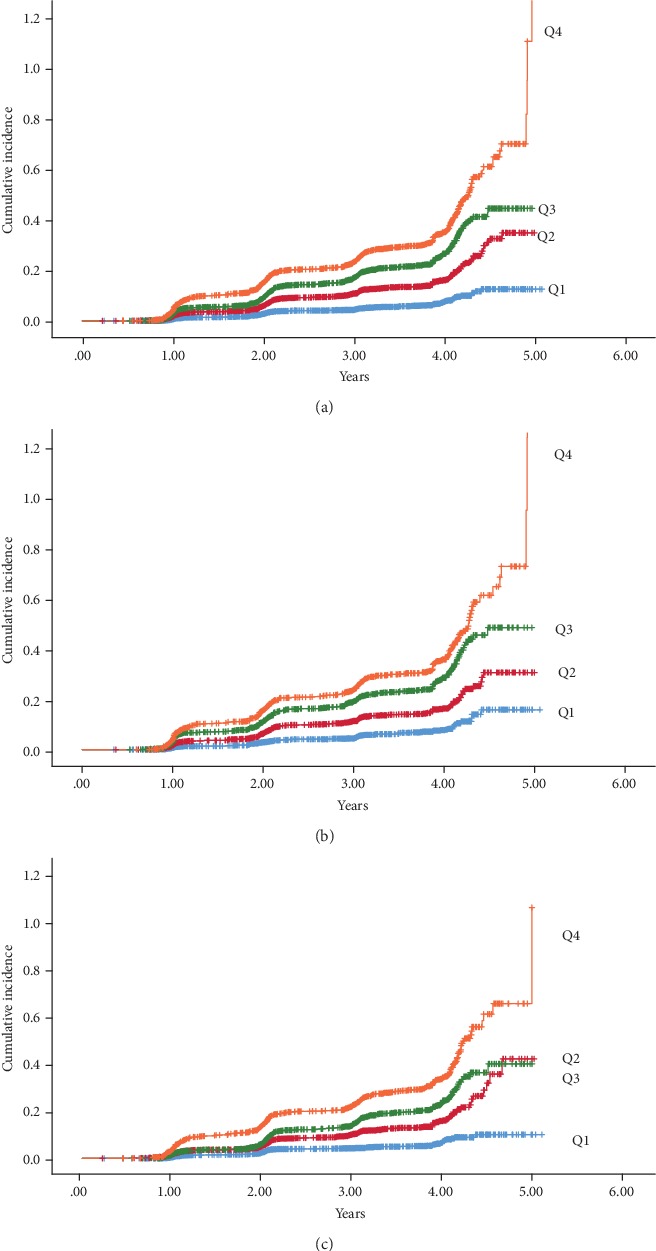
Cumulative incidence of nonalcoholic fatty liver disease (NAFLD) in the nonobese participants. (a) Cumulative incidence of NAFLD of the whole population stratified by mean arterial pressure (MAP). (b) Cumulative incidence of NAFLD of 8,472 men stratified by MAP. (c) Cumulative incidence of NAFLD of 7,681 women stratified by MAP. Q1 (58 to <80 mmHg), Q2 (80 to <88 mmHg), Q3 (88 to <96 mmHg), and Q4 (96 to ≤142 mmHg).

During a mean follow-up of 2.80 years, 2,321 participants developed new-onset NAFLD (Table 3). Overall, the cumulative incidence of NAFLD was 14.37 (13.68-15.06) and the incidence rate was 513.17 per 10,000 person-years. The cumulative incidence of NAFLD for the whole population or gender groups gradually increased with the quartiles of MAP (all *P* < 0.001). In the Q4 of MAP, the cumulative incidence of NAFLD for the whole population, male, and female reached up to 6.22 (5.75-6.70), 6.70 (6.21-7.19), and 5.69 (5.24-6.14), respectively. Besides, both incidence rate and cumulative incidence were higher in male than in female.  Figure 1 shows the cumulative incidence of NAFLD stratified by MAP.

### 3.3. The Association of MAP with the Incident NAFLD

To evaluate the association of MAP with the incident NAFLD, Cox's proportional hazard regression analyses were applied.  Table 4 summarizes the specific results of the univariate cox analysis and multivariate cox analysis. There was a positive association of MAP with NAFLD in the univariate cox analysis (*P* for trend < 0.001). As compared with Q1, the HR for NAFLD was 2.273 (1.921-2.689), 3.613 (3.075-4.245), and 5.083 (4.354-5.934) for Q2, Q3, and Q4, respectively. Then, a multivariate cox analysis was performed by including variables with a *P* value less than 0.2 in the univariate cox analysis, after adjustment for age, gender, BMI, SBP, DBP, ALP, ALT, AST, GLB, BUN, Cr, UA, FPG, TG, TC, TG, HDL-C, and LDL-C and the positive association of MAP with incident NAFLD remained significant (*P* for trend < 0.001). Similarly, compared with Q1, the multivariate HR for incident NAFLD was diminished but remained significant in Q2, Q3, and Q4, and the specific values for Q2, Q3, and Q4 were as follows: 1.328 (1.072-1.647), 1.625 (1.276-2.069), and 1.697 (1.231-2.340).

### 3.4. Subgroup Analysis by Gender

In order to verify the robustness of the combined effect of gender and MAP on incident NAFLD, the HR of incident NAFLD stratified into 2 groups of gender (male and female) is presented in Table 5. For female, after further adjustment for age, BMI, SBP, DBP, ALP, ALT, AST, GLB, BUN, Cr, UA, FPG, TG, TC, TG, HDL-C, and LDL-C, the risk of developing NAFLD gradually increased with quartiles of the MAP (*P* for trend = 0.002). The respective HR for NAFLD in Q2, Q3, and Q4 of MAP were 1.760 (1.276-2.429), 2.080 (1.433-3.019), and 2.377 (1.452-3.890), compared with female in the Q1 of MAP. In contrast, MAP was not associated with the risk of developing NAFLD in male (*P* > 0.05).

### 3.5. ROC Analysis of MAP and Risks for NAFLD

**Figure 2:**
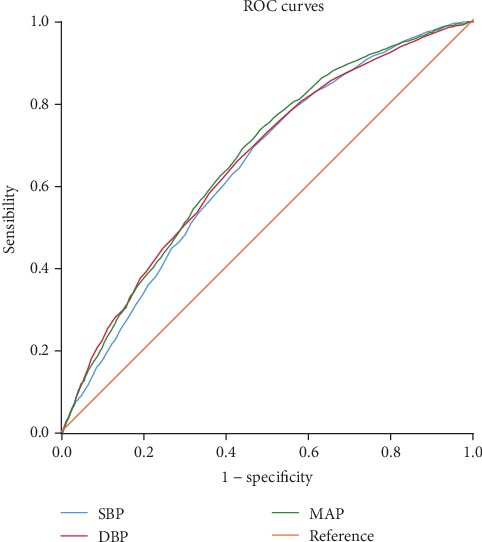
ROC curves for systolic blood pressure (SBP), diastolic blood pressure (DBP), and mean arterial pressure (MAP) for predicting nonalcoholic fatty liver disease in the 8,472 men.

**Figure 3:**
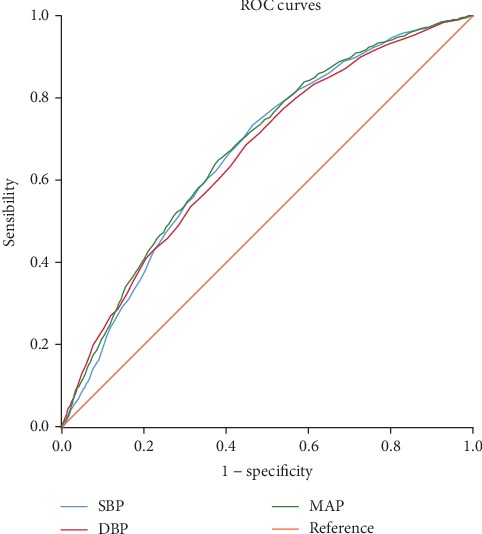
ROC curves for systolic blood pressure (SBP), diastolic blood pressure (DBP), and mean arterial pressure (MAP) for predicting nonalcoholic fatty liver disease in the 7,681 women.

As shown in Figures 2 and 3, SBP, DBP, and MAP were significant predictors for future risk of NAFLD in the group of male or female (all *P* < 0.05). The area under the ROC curves and their 95% confidence interval for male and female are shown in Table 6, respectively. And MAP had a larger area under the ROC curves than SBP or DBP. Additionally, the optimal cutoff points of MAP to predict NAFLD in male and female were 88 mmHg (with the sensitivity of 0.746 and specificity of 0.508) and 89 mmHg (with the sensitivity of 0.649 and specificity of 0.619), respectively.

## 4. Discussion

In this present study, we have demonstrated that MAP was one of the independent risk factors for incident NAFLD among nonobese participants with normal LDL-C levels in China. In subgroup analysis, although the incidence rate of NAFLD was significantly higher in male than female, the positive association between MAP and the incident NAFLD remained statistically significant in female, but not in male. Further, the areas under the ROC curves indicated that MAP was slightly superior to SBP or DBP for predicting NAFLD in the nonobese Chinese with normal LDL-C levels. Our results may be helpful for clinicians to identify subjects that are at a much higher risk for developing NAFLD.

Although individuals with NAFLD tend to be obese, quite a few NAFLD are nonobese population [11, 14], and unexpectedly, the proportion of nonobesity in NAFLD reached up to 75% in India [15]. The nonobese population with normal LDL-C levels in China had a 14.37% chance of developing NAFLD during five years of follow-up. Thus, it is quite necessary to determine a parameter that is reproducible, easily obtained, reliable, and practical for predicting the development of NAFLD in nonobese population with normal LDL-C. A bidirectional relationship between NAFLD and hypertension has been found by a prospective study [16]. That is to say, NAFLD can increase the risk of developing hypertension, whereas hypertension also increases the risk of developing NAFLD. Meanwhile, research achievements that elevated SBP or DBP which is independently correlated with an increased risk of the incident NAFLD in general population emerge in endlessly recent years [7–10]. Interestingly, two of the studies indicated elevated blood pressure is associated with incident NAFLD in subjects without hypertension [8, 10]. One study by Qian et al. demonstrated that both SBP and DBP are independent risk factors for incident NAFLD in nonhypertensive subjects [10], while Wu et al. argued that only the elevated SBP independently increases the risk of the incidence of NAFLD in subjects with normal SBP [8]. However, as far as we know, no data is available with respect to the correlation of MAP with incident NAFLD, not to mention a comparison of MAP with SBP or DBP for the prediction of NAFLD in nonobese population. We are the first to show that the elevated MAP independently increased the risk of the incident NAFLD, with an adjusted HR (Q4 versus Q1) of 1.697 (95% CI 1.231-2.340). After stratifying by gender, the adjusted HR increased to 2.377 (95% CI 1.452-3.890) in women. In addition, compared with SBP or DBP, MAP did slightly better to predict NAFLD in the nonobese Chinese with normal LDL-C levels, with optimal cutoff points of 88 mmHg in male and 89 mmHg in female.

After dividing the subjects into 2 groups of male and female, we found that male was at more risk of incident NAFLD than female. A previous study in Korea indicated that male accounts for the majority of nonobese subjects with NAFLD, but this proportion is relatively lower than obese subjects with NAFLD [17]. To some extent, the following reasons might be used to explain the gender difference of NAFLD. As shown in Table 2, other risk factors like age, BMI, blood pressure, FPG, and TG were higher in male than in female, and conversely, the levels of HDL-C decreased in male. Moreover, endogenous estradiol could play a protective role in the development of NAFLD [18].

The specific mechanism about the relationship between MAP and the incident NAFLD remains unclear. Considering that MAP is a combination of two components of BP: SBP and DBP, the proposed pathophysiologic mechanisms like insulin resistance, sympathetic nervous system activity, and arterial stiffness which are in regard to the relationship between increased BP and NAFLD [4] may serve as possible explanations that MAP independently increased the risk of incident NAFLD. For example, insulin resistance increases BP through enhancing salt absorption and activating the sympathetic nervous system [19], and hypertension is a predictor of insulin resistance in turn [20, 21], whereas insulin resistance leads to liver endothelial dysfunction and then promotes NAFLD [20, 22]. Furthermore, as a steady component, MAP is strongly associated with an increased risk of cardiovascular disease and diabetes [23, 24]. Therefore, in the nonobese population with normal LDL-C levels, MAP could be applied to identify subjects who are more susceptible to NAFLD.

There are some limitations of our study. Firstly, ultrasonography is a common way to diagnose NAFLD in epidemiological surveys but hardly assesses the severity of NAFLD. Secondly, insulin resistance plays an important role in developing NAFLD among the nonobese, but insulin levels and insulin resistance were not examined in the initial study. Thirdly, some important variables like lifestyle, smoking, and nutrition are unavailable. Lastly, the information about changes in anthropometric parameters over time and the status of appearance of other components of metabolic syndrome during the study period were also unavailable.

## 5. Conclusion

Increased MAP is independently associated with NAFLD in nonobese female with normal LDL-C levels, but not in nonobese male, which behaves slightly better in NAFLD prediction. MAP may be clinically useful in identifying people at a higher risk of developing NAFLD.

## Figures and Tables

**Table 1:** Baseline characteristics of 16,153 participants by mean arterial pressure quartiles.

Characteristics	58 ≤ Q1 < 80 mmHg	80 ≤ Q2 < 88 mmHg	88 ≤ Q3 < 96 mmHg	96 ≤ Q4 ≤ 142 mmHg	*P* value
Number (%)	3,806(23.6)	4,414 (27.3)	3,744 (23.2)	4,189 (25.9)	
Age (years)	41.66 ± 14.32	42.32 ± 14.64	43.29 ± 15.04	45.55 ± 15.50	<0.001
Male, *N* (%)	1,817 (47.7)	2,247 (50.9)	2,049 (54.7)	2,359 (56.3)	<0.001
BMI (kg/m^2^)	20.45 ± 1.94	21.15 ± 1.98	21.67 ± 1.95	22.20 ± 1.90	<0.001
SBP (mmHg)	102.53 ± 6.79	114.29 ± 6.41	124.08 ± 7.69	141.05 ± 13.11	<0.001
DBP (mmHg)	60.88 ± 3.91	68.67 ± 3.51	75.43 ± 4.11	85.68 ± 7.21	<0.001
ALP (U/L)	65.22 ± 21.44	70.20 ± 21.60	73.79 ± 25.16	77.92 ± 22.65	<0.001
ALT (U/L)	16.71 ± 12.14	19.59 ± 18.48	21.05 ± 15.26	21.91 ± 17.51	<0.001
AST (U/L)	20.79 ± 7.73	22.34 ± 9.80	23.48 ± 8.11	24.82 ± 10.88	<0.001
ALB (g/L)	44.01 ± 2.49	44.46 ± 2.71	44.65 ± 2.75	44.46 ± 2.83	<0.001
GLB (g/L)	29.32 ± 3.55	29.33 ± 3.92	29.36 ± 3.78	29.95 ± 4.11	<0.001
TB (*μ*mol/L)	11.50 ± 4.50	12.07 ± 5.24	12.28 ± 4.89	12.53 ± 5.00	<0.001
BUN (mmol/L)	4.25 ± 1.13	4.46 ± 1.24	4.64 ± 1.39	4.90 ± 1.58	<0.001
Cr (mmol/L)	70.62 ± 15.49	76.21 ± 17.82	81.14 ± 24.78	85.66 ± 36.40	<0.001
UA (*μ*mol/L)	244.36 ± 72.24	270.37 ± 81.13	291.36 ± 86.11	311.70 ± 88.33	<0.001
FPG (mmol/L)	4.91 ± 0.52	5.03 ± 0.63	5.18 ± 0.80	5.44 ± 0.98	<0.001
TC (mmol/L)	4.47 ± 0.71	4.57 ± 0.72	4.67 ± 0.75	4.78 ± 0.76	<0.001
TG (mmol/L)	1.03 ± 0.54	1.21 ± 0.78	1.38 ± 0.93	1.57 ± 1.18	<0.001
HDL-C (mmol/L)	1.51 ± 0.35	1.48 ± 0.36	1.44 ± 0.37	1.42 ± 0.37	<0.001
LDL-C (mmol/L)	2.16 ± 0.46	2.24 ± 0.46	2.30 ± 0.46	2.35 ± 0.46	<0.001
Duration of follow up (years)	2.89 ± 1.11	2.76 ± 1.13	2.82 ± 1.14	2.75 ± 1.19	<0.001
NAFLD (%)	191 (5.0)	473 (10.7)	652 (17.4)	1,005 (24.0)	<0.001

Note: continuous variables were described by mean ± standard deviation, and categorical variables were presented by number (percentage). Abbreviations: BMI = body mass index; SBP = systolic blood pressure; DBP = diastolic blood pressure; ALP = alkaline phosphatase; ALT = alanine aminotransferase; AST = aspartate aminotransferase; ALB = albumin; GLB = globulin; TB = total bilirubin; BUN = blood urea nitrogen; Cr = creatinine; UA = uric acid; FPG = fasting plasma glucose; TC = total cholesterol; TG = triglyceride; HDL-C = high density lipoprotein cholesterol; LDL-C = low-density lipoprotein cholesterol; NAFLD = nonalcoholic fatty liver disease.

**Table 2:** Baseline characteristics of 16,153 participants stratified by gender.

Characteristics	Female	Male	*P* value
Number (%)	7,681 (47.6)	8,472 (52.4)	
Age (years)	40.37 ± 12.72	45.82 ± 16.31	<0.001
BMI (kg/m^2^)	21.26 ± 2.02	21.50 ± 2.07	<0.001
SBP (mmHg)	119.62 ± 16.59	121.74 ± 16.77	<0.001
DBP (mmHg)	72.14 ± 10.18	73.42 ± 10.47	<0.001
MAP (mmHg)	87.96 ± 11.46	89.53 ± 11.67	0.139
ALP (U/L)	71.06 ± 22.49	73.41 ± 23.77	<0.001
ALT (U/L)	19.68 ± 18.19	20.37 ± 14.86	0.022
AST (U/L)	22.83 ± 9.99	23.21 ± 9.08	0.031
ALB (g/L)	44.28 ± 2.63	44.51 ± 2.77	<0.001
GLB (g/L)	29.56 ± 3.86	29.44 ± 3.87	0.068
TB (*μ*mol/L)	11.87 ± 4.72	12.34 ± 5.13	<0.001
BUN (mmol/L)	4.52 ± 1.36	4.61 ± 1.37	<0.001
Cr (mmol/L)	76.55 ± 25.45	80.24 ± 25.79	<0.001
UA (*μ*mol/L)	271.72 ± 84.41	287.17 ± 86.64	<0.001
FPG (mmol/L)	5.12 ± 0.80	5.16 ± 0.76	0.002
TC (mmol/L)	4.62 ± 0.74	4.62 ± 0.75	0.971
TG (mmol/L)	1.25 ± 0.79	1.35 ± 1.01	<0.001
HDL-C (mmol/L)	1.48 ± 0.35	1.45 ± 0.36	<0.001
LDL-C (mmol/L)	2.26 ± 0.46	2.27 ± 0.47	0.125
Duration of follow-up (years)	2.85 ± 1.12	2.76 ± 1.16	<0.001
NAFLD (%)	1,027 (13.4)	1,294 (15.3)	0.001

Note: continuous variables were described by mean ± standard deviation, and categorical variables were presented by number (percentage). Abbreviations: BMI = body mass index; SBP = systolic blood pressure; DBP = diastolic blood pressure; MAP = mean arterial pressure; ALP = alkaline phosphatase; ALT = alanine aminotransferase; AST = aspartate aminotransferase; ALB = albumin; GLB = globulin; TB = total bilirubin; BUN = blood urea nitrogen; Cr = creatinine; UA = uric acid; FPG = fasting plasma glucose; TC = total cholesterol; TG = triglyceride; HDL-C = high density lipoprotein cholesterol; LDL-C = low-density lipoprotein cholesterol; NAFLD = nonalcoholic fatty liver disease.

**Table 3:** Incidence rate of NAFLD stratified by mean arterial pressure.

Group	Number	Number of NAFLD	Cumulative incidence (95% CI)	Per 10,000 person-years
Total	16153	2321	14.37 (13.68-15.06)	513.17
Q1	3806	191	1.18 (0.97-1.39)	173.66
Q2	4414	473	2.93 (2.60-3.26)	388.26
Q3	3744	652	4.04 (3.65-4.42)	617.54
Q4	4189	1005	6.22 (5.75-6.70)	872.41
*P* value for log-rank test	—	—	<0.001	—
Male	8472	1294	15.27 (14.57-15.99)	553.40
Q1	1817	98	1.16 (0.95-1.37)	188.58
Q2	2247	244	2.88 (2.55-3.21)	397.76
Q3	2049	384	4.53 (4.12-4.94)	681.49
Q4	2359	568	6.70 (6.21-7.19)	888.49
*P* value for log-rank test	—	—	<0.001	—
Female	7681	1027	13.37 (12.70-14.04)	469.15
Q1	1989	93	1.21 (1.00-1.43)	160.68
Q2	2167	229	2.98 (2.65-3.31)	377.41
Q3	1695	268	3.49 (3.13-3.85)	543.34
Q4	1830	437	5.69 (5.24-6.14)	852.85
*P* value for log-rank test	—	—	<0.001	—

Abbreviations: CI = confidence interval; NAFLD = nonalcoholic fatty liver disease.

**Table 4:** Univariate Cox regression analysis for incident nonalcoholic fatty liver disease.

Characteristics	Univariate analysis		Multivariate analysis	
	HR (95% CI)	*P* value	HR (95% CI)	*P* value
Q1	Ref		Ref	
Q2	2.273 (1.921-2.689)	<0.001	1.328 (1.072-1.647)	0.010
Q3	3.613 (3.075-4.245)	<0.001	1.625 (1.276-2.069)	<0.001
Q4	5.083 (4.354-5.934)	<0.001	1.697 (1.231-2.340)	0.001
*P* for trend		<0.001		0.001
Age	1.006 (1.004-1.009)	<0.001	1.008 (1.004-1.011)	0.000
Gender	1.181 (1.088-1.282)	<0.001	0.933 (0.850-1.025)	0.147
BMI	1.815 (1.765-1.866)	<0.001	1.535 (1.485-1.587)	<0.001
SBP	1.023 (1.021-1.025)	<0.001	0.993 (0.988-0.997)	0.002
DBP	1.046 (1.042-1.050)	<0.001	1.007 (0.999-1.016)	0.089
ALP	1.008 (1.007-1.009)	<0.001	1.005 (1.003-1.007)	<0.001
ALT	1.007 (1.006-1.008)	<0.001	1.014 (1.011-1.017)	<0.001
AST	1.009 (1.007-1.011)	<0.001	0.977 (0.968-0.985)	<0.001
ALB	1.005 (0.988-1.021)	0.586		
GLB	1.008 (0.997-1.020)	0.166	0.999 (0.987-1.011)	0.860
TB	0.998 (0.989-1.008)	0.695		
BUN	0.933 (0.903-0.963)	<0.001	0.828 (0.796-0.862)	<0.001
Cr	1.005 (1.004-1.005)	<0.001	1.002 (1.001-1.004)	<0.001
UA	1.005 (1.005-1.006)	<0.001	0.999 (0.999-1.000)	0.035
FPG	1.297 (1.268-1.327)	<0.001	1.210 (1.170-1.251)	<0.001
TC	1.300 (1.238-1.365)	<0.001	0.776 (0.690-0.872)	<0.001
TG	1.204 (1.192-1.216)	<0.001	1.234 (1.178-1.293)	<0.001
HDL-C	0.278 (0.245-0.316)	<0.001	0.797 (0.664-0.956)	0.014
LDL-C	1.950 (1.774-2.143)	<0.001	1.850 (1.551-2.207)	<0.001

Note: ALB and TB were not included into multivariate analysis for their *P* value more than 0.2 in the univariate cox analysis. Abbreviations: BMI = body mass index; SBP = systolic blood pressure; DBP = diastolic blood pressure; ALP = alkaline phosphatase; ALT = alanine aminotransferase; AST = aspartate aminotransferase; ALB = albumin; GLB = globulin; TB = total bilirubin; BUN = blood urea nitrogen; Cr = creatinine; UA = uric acid; FPG = fasting plasma glucose; TC = total cholesterol; TG = triglyceride; HDL-C = high density lipoprotein cholesterol; LDL-C = low-density lipoprotein cholesterol; HR = hazard ratio; CI = confidence interval.

**Table 5:** Univariate and multivariate Cox regression analysis for incident nonalcoholic fatty liver disease, stratified by gender.

	Univariate analysis		Multivariate analysis	
	HR (95% CI)	*P* value	HR (95% CI)	*P* value
Male				
Q1	Ref		Ref	
Q2	2.133 (1.687-2.697)	<0.001	1.042 (0.780-1.391)	0.782
Q3	3.692 (2.957-4.609)	<0.001	1.321 (0.959-1.819)	0.088
Q4	4.792 (3.867-5.939)	<0.001	1.314 (0.857-2.015)	0.211
*P* for trend		<0.001		0.071
Female				
Q1	Ref		Ref	
Q2	2.416 (1.898-3.074)	<0.001	1.760 (1.276-2.429)	0.001
Q3	3.424 (2.704-4.335)	<0.001	2.080 (1.433-3.019)	<0.001
Q4	5.331 (4.262-6.669)	<0.001	2.377 (1.452-3.890)	0.001
*P* for trend		<0.001		0.002

Abbreviations: HR = hazard ratio; CI = confidence interval.

**Table 6:** The area under the ROC curves and their 95% confidence interval for male and female.

	Area under the curve (95% confidence interval)	
	Male	Female
Systolic blood pressure	0.648 (0.633-0.663)	0.668 (0.652-0.685)
Diastolic blood pressure	0.659 (0.644-0.675)	0.663 (0.646-0.680)
Mean arterial pressure	0.666 (0.651-0.681)	0.676 (0.660-0.693)

## Data Availability

The data that support the findings of this study are openly available in Dryad repository at 10.5061/dryad.1n6c4.
